# Phytochemical Composition and Bioactive Effects of *Salvia africana*, *Salvia officinalis ‘Icterina’* and *Salvia mexicana* Aqueous Extracts

**DOI:** 10.3390/molecules24234327

**Published:** 2019-11-27

**Authors:** Andrea F. Afonso, Olívia R. Pereira, Ângela Fernandes, Ricardo C. Calhelha, Artur M. S. Silva, Isabel C.F.R. Ferreira, Susana M. Cardoso

**Affiliations:** 1 QOPNA & LAQV-REQUIMTE, Department of Chemistry, University of Aveiro, 3810-193 Aveiro, Portugal; andrea@ipb.pt (A.F.A.); artur.silva@ua.pt (A.M.S.S.); 2 Public Health Laboratory of Bragança, Local Health Unit, Rua Eng. Adelino Amaro da Costa, 5300-146 Bragança, Portugal; 3 Centro de Investigação de Montanha (CIMO), Instituto Politécnico de Bragança, Campus de Santa Apolónia, 5300-253 Bragança, Portugal; oliviapereira@ipb.pt (O.R.P.); afeitor@ipb.pt (Â.F.); calhelha@ipb.pt (R.C.C.);

**Keywords:** *Salvia*, phenolic compounds, high performance liquid chromatography, antioxidant, anti-inflammatory, cytotoxicity, antibacterial

## Abstract

In the present study, aqueous extracts of *Salvia africana*, *Salvia officinalis ‘Icterina’* and *Savia mexicana* origin were screened for their phenolic composition and for antibacterial, antioxidant, anti-inflammatory and cytotoxic properties. The three aqueous extracts contained distinct phenolic compounds, with *S. africana* presenting the highest total levels (231.6 ± 7.5 μg/mg). Rosmarinic acid was the dominant phenolic compound in all extracts, yet that of *S. africana* origin was characterized by the present of yunnaneic acid isomers, which overall accounted for about 40% of total phenolics. In turn, *S. officinalis ‘Icterina’* extract presented glycosidic forms of apigenin, luteolin and scuttelarein, and the one obtained from *S. mexicana* contained several simple caffeic acid derivatives. *S. africana* aqueous extract exhibited high antioxidant potential in four methods, namely the DPPH^•^ (2,2-diphenyl-1-picrylhydrazyl) scavenging ability, iron-reducing power, inhibition of β-carotene bleaching and of thiobarbituric acid reactive substances (TBARS), for which EC_50_ values were equal or only 1.3–3.1 higher than those of the standard compounds. Moreover, this extract was able to lower the levels of nitric oxide (NO) production in lipopolysaccharide (LPS)-activated RAW 264.7 macrophages (EC_50_ = 47.8 ± 2.1 μg/mL). In addition, the three sage aqueous extracts showed promising cytotoxic effect towards hepatocellular HepG2, cervical HeLa, and breast carcinoma cells MCF-7. Overall this study highlights the potential of three little-exploited *Salvia* species, with commercial value for applications in food or pharmaceutical industries.

## 1. Introduction

**Figure 1:**
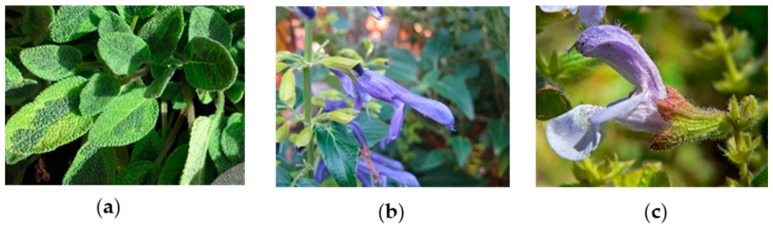
*Salvia* plants: (**a**) *S. officinalis ‘Icterina’*; (**b**) *S. mexicana*; (**c**) *S. africana*; retrieved from: https://pt.wikipedia.org/.

*Salvia* plants are distributed worldwide and are particularly prevalent in tropical and temperate zones in the Mediterranean region of Europa, South-East Asia and Central and South America [1,2]. Due to their claimed health effects, many *Salvia* species are considered promising for application in diverse areas, including the food, pharmaceutical and cosmetic industries [3,4]. Among the bioactive components of *Salvia*, terpenes, phenolic acids and flavonoids have been highlighted. In fact, on the basis of *in vitro* and *in vivo* studies, the essential oils and phenolic rich extracts of widely distributed species such as *Salvia officinalis*, *Salvia miltiorrhiza* and *Salvia sclarea* are assumed to exert potent antimicrobial, antioxidant and anti-inflammatory properties, among other beneficial effects [4,5,6,7]. Importantly, even though for the last few years there has been an increasing number of research works aiming to screen the biological properties of *Salvia* plants, many species and/or cultivars including *S. officinalis ‘Icterina’*, *Salvia mexicana* and *Salvia africana* (Figure 1) remain poorly explored. *S. officinalis ‘Icterina’* is a cultivar of *S. officinalis* characterized by yellow green variegated leaves [8], but in opposition to the last species, little is known about its chemical composition and potential bioactivities. Likewise, *S. mexicana* (native of central Mexico [9]) is mainly known for ornamental purposes while chemical elucidation has been only focused on its essential oils [10]. In turn, *S. africana* (native of Africa) has long been used by african people to flavour food and as a medicinal plant to treat cold, flu, coughs and women’s ailments [11,12] and its essential oil composition has been revealed [13,14,15,16], but knowledge of other bioactive metabolites remains unknown. Given the lack of information, the present study constitutes the first attempt to elucidate the phenolic composition as well as the antioxidant, anti-inflammatory, cytotoxic and antibacterial activities of aqueous extracts of *S. officinalis ‘Icterina’*, *S. mexicana* and *S. africana.*

## 2. Results and Discussion

### 2.1. Phenolic Compounds in *Salvia* Aqueous Extracts

The mass yield of the three *Salvia* aqueous extracts ranged from 19–22%, being also similar to those previously reported for *S. elegans*, *S. greggii* and *S. officinalis* L. [17]. Still, regardless of the close mass yields among the three target aqueous extracts, the total phenolic compound content in the *S. africana* extract was clearly superior than those of the two other species (350.6 ± 14.9 μg gallic equivalents (GAE)/mg vs. 229.0 ± 44.0 and 158.9 ± 38.0 μg GAE/mg) (Table 1). Naturally, differences were reflected in the levels of the main phenolic component, i.e rosmarinic acid (RT 19.0 min, UV_max_ at 290 and 328 nm, [M − H]^−^ at *m*/*z* 359 → 161, 179, 197, 223), which accounted for 77.0 ± 3.6 μg/mg in *S. africana* aqueous extract and 52.7 ± 0.5 and 29.4 ± 0.6 μg/mg in those of *S. officinalis ‘Icterina’* and *S. mexicana* origins, respectively. Moreover, the three extracts were also distinguishable in relation to their remaining phenolic components. *S. africana* was characterized by the presence of several other complex caffeic acid derivatives, particularly by distinct yunnaneic acid E isomers (UV_max_ at 268, 274, 276, 279, [M − H]^−^ at *m*/*z* 571), which overall accounted for 74 μg/mg extract (equivalent to about 32% of the total quantified phenolics). It also contained minor quantities of yunnaneic acid F (RT 14.3 min, [M−H]^−^ at *m*/*z* 597→ 579, 355, 312, 295, 197, 179) and yunnaneic acid D (RT 15.6, and 15.9 min, [M − H]^−^ at *m*/*z* 539), three caffeoyl rosmarinic acid isomers (RT 19.2 min, 19.5 min and 21.4 min, [M − H]^−^ at *m*/*z* 537), salvianolic acid K (RT 15.1 min, [M − H]^−^ at *m*/*z* 555→ 313, 357), salvianolic acid B (RT 18.6 min, [M − H]^−^ at *m*/*z* 717→ 555, 519, 475, 357), and sagerinic acid (RT 17.6 min, [M − H]^−^ at *m*/*z* 719→ 359, 539, 521, 341).

Although some complex caffeic derivatives were also found in *S. mexicana* extract (yunnaneic acids E and D), this was distinguishable from the others due to the presence of several simple phenolic derivatives such as isomers of caffeoylquinic acid (RT 5.0 min, 8.8 min and 9.5 min, [M − H]^−^ at *m*/*z* 353 → 191), caftaric acid (RT 5.3 min, [M − H]^−^ at *m*/*z* 311 → 149, 179), caffeoyl malic acid (RT 12.3 min, [M − H]^−^ at *m*/*z* 295 → 207, 179, 133, 135), sagerinic acid (RT 17.6 and 19.8 min, [M − H]^−^ at *m*/*z* 719) and the flavanone hesperidin (RT 18.7 min, [M − H]^−^ at *m*/*z* 609 → 301). In turn, *S. officinalis ‘Icterina’* aqueous extract was rich in flavone glycosides (accounting for about 40% of total quantified phenolic compounds), mainly represented by luteolin-*O*-glucuronide (RT 16.1 min, [M − H]^−^ at *m*/*z* 461, 18.2 ± 0.4 μg/mg extract), apigenin-*O*-glucuronide (RT 18.4 min, [M − H]^−^ at *m*/*z* 445→ 269, 175, 32.8 ± 0.5 μg/mg extract) and scutellarein-*O*-glucuronide (RT 15.9 min, [M − H]^−^ at *m*/*z* 461→ 285, 9.7 ± 0.2 μg/mg extract). Note that regardless of whether the major compounds herein found in *S. officinalis ‘Icterina’* aqueous extract (rosmarinic acids, luteolin-*O*-glucuronide, apigenin-*O*-glucuronide and scutellarein-*O*-glucuronide) correspond to those previously reported for *S. officinalis*, the superior levels of rosmarinic acid found in the first one (52.7 ± 0.5 vs. 28.3 ± 0.6 μg/mg extract [17]) suggests that this cultivar may be richer in rosmarinic acid compared to *S. officinalis*.

### 2.2. Bioactive Properties of Salvia Aqueous Extracts

#### 2.2.1. Antibacterial Activity

The inhibitory capacities of the three *Salvia* aqueous extracts towards the Gram-positive bacteria *Staphylococcus aureus* and *Staphylococcus epidermidis*, and the Gram-negative bacteria *Salmonella typhimurium*, *Escherichia coli* and *Pseudomonas aeruginosa,* are summarized in Table 2. As reported in literature for phenolic-rich extracts in general [18,19], Gram-positive bacteria showed higher sensibility to the *Salvia* aqueous extracts compared to the Gram-negative pathogens, with minimum inhibitory concentration (MIC) and minimum bactericidal concentration (MBC) values of *S. aureus* and *S. epidermidis* in the range of 0.63–4.75 and 0.94–9.50 mg/mL, respectively. In fact, for these two Gram-positive strains, *S. africana* aqueous extract (i.e., the richest in phenolic compounds) was the most promising, with MIC and MBC values of 0.63–1.25 mg/mL and 1.25 mg/mL, respectively.

Notably, among the three aqueous extracts, that of *S. officinalis ‘Icterina’* had a superior antibacterial capacity against Gram-negative bacteria (both MIC and MBC of 3.75 or 7.5 mg/mL), a fact that may possibly be correlated with its unique richness in flavones, although we cannot rule out a possible contribution of non-phenolic compounds from the extract. As for the two other aqueous extracts, that of *S. africana* origin exhibited antibacterial ability towards *S. typhimurium* and *E. coli*, but was ineffective against *P. aeruginosa* and in turn, *S. mexicana* presented a modest antimicrobial effect against *E. coli* and *P. aeruginosa* and required concentrations above 9.5 mg/mL to be effective against *S. typhimurium*.

To our knowledge, literature data reporting the antimicrobial potential of polar extracts of the three *Salvia* species under study are restricted to *S. officinalis ‘Icterina’*. More specifically, based on the agar disc diffusion assay, methanolic extracts of *S. officinalis ‘Icterina’* were shown to inhibit the grown of Gram-positive and Gram-negative bacteria, for which the authors obtained inhibition hales about 9–10 mm at 143 mg/mL [8]. Yet, as no information was given about MIC values, a direct comparison to our results is not possible. Moreover, among studies that evaluated the antibacterial potential of *Salvia* polar extracts, only a few determined the MBC value, making it difficult to perceive their microbicidal potential. However, considering the previous MIC and MBC values reported by our group for aqueous extracts of *Salvia apiana* and *Salvia farinacea* var. *victoria blue* towards the same pathogens, we can conclude that among the six *Salvia* species, *S. apiana* is the most promising regarding antimicrobial effects (both MIC and MBC in the range 0.69–2.75 mg/mL) [20].

Also noteworthy is that the antibacterial data found herein exceeded those previously reported for *Salvia* extracts of distinct origins using the microdilution method. In particular, aqueous extracts of *Salvia ringens* [19] and *Salvia amplexicaulis* [18], both rich in kaempferol glycosides and/or in caffeic and rosmarinic acids, inhibited *S. aureus* strains (MIC of 15 and 40 mg/mL, respectively) and Gram-negative bacteria (MIC of 20 to ≥ 50 mg/mL). In turn, Toplan et al. [21] found promising results when testing a methanolic extract of *Salvia veneris* (rich in rosmarinic acid and luteolin glucoside), which exhibited inhibitory effect against strains of *S. aureus* and *S. epidermidis* (MIC value of 0.25 and 2.0 mg/mL, respectively), and also against *P. aeruginosa* and *S. typhimurium* (MIC of 0.5 and 2.0 mg/mL, respectively) [21]. In their research, the authors found that essential oil showed relatively weak antimicrobial activity compared to the methanol extract, underlining the antibacterial role of polar extracts.

#### 2.2.2. Antioxidant Activity

The results from the antioxidant evaluation, as measured by the capacity to scavenge 2,2-diphenyl-1-picrylhydrazyl (DPPH^•^), to reduce ferric iron and to inhibit peroxidation events (β-carotene bleaching and thiobarbituric acid reactive substances (TBARS) assays), are summarized in Table 3.

Amongst the three plant aqueous extracts, that of *S. africana* was the most active, exhibiting similar potency to that of standard compounds in DPPH^•^, ferric reducing power and TBARS methods, a fact that may be associated to its richness in phenolic compounds, as previously described for plants extracts. In fact, methanolic extracts of *S. officinalis* [22,23], rich in glycosidic flavones, rosmarinic acids and caffeic acid derivatives, together with other polar extracts of several *Salvia* species that had high content in flavonoid compounds (e.g., luteolin, apigenin, quercetin and their glycosides) [18,22,24,25,26], were shown to be promising antioxidant agents.

Concerning the two other sage aqueous extracts under study, the results varied among the assays, possibly as a reflect of their different compositions and the distinct mechanisms of action involved. In fact, although they showed the same ability to scavenge the DPPH^•^ and to reduce Fe^3+^, that of *S. officinalis ‘Icterina’* was significantly more efficient in protecting against lipid peroxidation events in β-carotene bleaching inhibition test. Yet, overall, one must highlight the high potential of the three *Salvia* extracts regarding the prevention of oxidative-related events, as evidenced by their EC_50_ values which were in the same order or, in maximum, 4 times over those of trolox.

Also of note, the results herein gathered surpassed that previously described for a *S. africana* extract obtained with methanol:chloroform (1:1), for which DPPH^•^ EC_50_ value was reported to be 33.4 ± 3.73 μg/mL and equivalent to 13.3-fold that of the positive control trolox [14]. As far as we know, there is no literature data regarding the antioxidant abilities of aqueous extracts of *S. mexicana* and *S. officinalis ‘Icterina’* origin. Yet, it is curious that the potential of *S. officinalis ‘Icterina’* aqueous extract to scavenge DPPH^•^ was 3 times above than that of *S. officinalis* (EC_50_ of 10.4 vs. 34.8 μg/mL, respectively) [17], suggesting that *‘Icterina’* cultivar may be more active than *S. officinalis* in scavenging free radicals.

#### 2.2.3. Anti-Inflammatory Activity

Due to its relevance in inflammatory events, the radical nitric oxide (NO) is considered to be a key target for anti-inflammatory agents [27]. In this sense, the ability of the three *Salvia* aqueous extracts to act as inhibitory agents of nitric oxide production by activated macrophages was assessed in the lipopolysaccharide (LPS)-stimulated RAW 264.7 macrophage cell model. As shown in Table 3, *S. africana* had the most promising activity, which corresponded to about 3-fold that of dexamethasone used as a reference compound (EC_50_ of 47.8 ± 2.1 μg/mL vs. 16.0 ± 1.0 μg/mL, respectively), while *S. officinalis ‘Icterina’* and *S. mexicana* showed close efficacies, albeit tending to be superior in the first one (EC_50_ values of 60.3 and 66.3 μg/mL, respectively), also following the same tendency as for their levels of phenolic compounds. Note that the ability of *Salvia* polar extracts to act towards NO has been scarcely studied. However, we have previously underlined the scavenging capacity of this radical by *S. officinalis*, *S. elegans* and *S. greggii* aqueous extracts, as estimated in chemical assay [17], as well as the efficacy of *S. apiana* and *S. farinacea* var. *victoria blue* to reduce NO production in the same macrophage cellular model (EC_50_ = 49.9 and 80.8 μg/mL, respectively) [20]. Also, commercial aqueous extracts of *S. miltiorrhiza* [28,29] and ethanolic extracts of *Salvia plebeia* [30] were previously shown to modulate levels of NO by activated RAW 264.7 macrophage cells, in a concentration-dependent manner, albeit for high EC_50_ values (of 200 μg/mL and higher than 500 μg/mL, respectively).

Notably, although no *in vivo* anti-inflammatory activity of the herein target plants were previously reported, several *Salvia* species were shown to act as anti-inflammatory agents. E.g., methanolic extracts obtained from sage species (*Salvia halophila*, *Salvia virgata*, *Salvia splendens*, *Salvia fruticosa* and *Salvia bicolor*), all rich in phenolic acids (caffeic, rosmarinic, *o*-coumaric, gallic acid), and/or flavonoids, particularly luteolin and apigenin in glyosidic or aglycone forms, were described to reduce carrageenan-induced paw edema in rat models [31,32,33,34]. Likewise, a decrease in the levels of cytokines IL-6, IL-1β and TNF-α were reported after oral administration of ethanolic extract from *S. sclarea* in a rat model of periodontal disease, a fact that authors have associated to its high content in rosmarinic acid [24]. Importantly, inhibition of production and mRNA expression of cytokines IL-6, IL-1β, and TNF-α in stimulated THP-1 macrophages were already demonstrated for lithospermic acid extracted from the root of *S. miltiorrhiza* [35], thus highlighting their central role on the anti-inflammatory properties of these plants.

#### 2.2.4. Cytotoxic Activity

The cytotoxic ability of the three *Salvia* extracts on tumor and non-tumor cell lines was carried out by the sulforhodamine B (SRB) test, which allows estimation of the number of metabolically active cells present in culture. Similarly to our results previously reported for *S. apiana* and *S. farinacea* var. *vitoria blue* [20], the present data confirms promising cytotoxic effects of *S. africana, S. officinalis ‘Icterina’* and *S. mexicana* aqueous extracts against hepatocellular carcinoma HepG2, cervical carcinoma HeLa and breast carcinoma MCF-7 cells, as well their tumor-selectivity as reflected by comparatively low GI_50_ values in the cancer cell lines compared to those of non-tumoral PLP2 cells (Table 3). Overall, the three extracts showed similar cytotoxicity (*p* > 0.05), except for the extract from *S. officinalis ‘Icterina’* against HeLa cells, which showed significantly lower activity than the others. As far as we are aware, the potential cytotoxic effect of the present target *Salvia* species has not been exploited before and since, our results represent the first alert for their potency to serve as anti-tumoral agents. Thus, although we cannot directly compare our results with others previously reported (once different extracts, controls and methods have been used), the results presented herein emphasizes the potential of sage polar extracts in tumoral cell inhibition. The cytotoxic effect of ethanolic extracts from leaves and roots of *S. officinalis* on HepG2 cells after 24 h of exposure was evaluated by Jiang et al. [36], showing half maximal inhibition concentration (IC_50_) values of 84.0 and 75.8 µg/mL, respectively. In that study, both extracts exhibited less anti-proliferation ability in normal human liver cells (WRL-68), with higher IC_50_ values (> 100 µg/mL) compared with the effect on HepG2 cells [36]. Similarly, Shahneh et al. [37] reported an inhibitory effect of a crude methanolic extract of *S. officinalis* against tumor cell lines, as reflected by the comparatively low IC_50_ values when exposed to MCF-7 cells (142 μg/mL), to that obtained towards Human Umbilical Vein Endothelial Cells (HUVEC, IC_50_ values exceeding 600 µg/mL).

## 3. Materials and Methods

### 3.1. Chemicals

Rosmarinic acid, the 7-*O*-glucoside derivatives of apigenin, luteolin and eriodictyol, caffeic acid, 5-*O*-caffeoylquinic acid, 4-*O*-hydroxybenzoic acid, salvianolic acid B, ferulic acid, and 4-*O*-coumaric acid were obtained from Extrasynthese (Genay Cedex, France). Gallic acid, nisin, ascorbic acid, trolox, sulforhodamine B (SRB), DPPH^•^ (2,2-diphenyl-1-picrylhydrazyl) radical, acetic acid, ellipticine, trypan blue, trichloroacetic acid (TCA), Tris, lipopolysaccharide (LPS), nisin, and butylated hydroxyanisole (BHA) were obtained from Sigma Chemical Co (St Louis, MO, USA). Folin-Ciocalteu reagent, Na_2_CO_3_, formic acid and ethanol were purchased from Panreac (Barcelona, Spain). *n*-hexane, methanol and acetonitrile with high-performance liquid chromatography (HPLC) purity were purchased from Lab-Scan (Lisbon, Portugal). Fetal bovine serum (FBS), l-glutamine, trypsin-ethylenediaminetetraacetic acid (EDTA), penicillin/streptomycin solution (100 U/mL and 100 mg/mL, respectively), RPMI 1640 and DMEM media were obtained from Hyclone (Logan, Utah, UT, USA). The Griess reagent system was purchased from Promega Corporation (Madison, WI, USA). Mueller-Hinton agar was purchased from VWR (Prolabo Chemicals, West Chester, PA, USA). Purified water was obtained from a Direct-Q^®^ water purification system (Merck Life Science, Darmstadt, Germany).

### 3.2. Plant Material

The *S. africana, S. officinalis ‘Icterina’* and *S. mexicana* species were collected from the fields of Coimbra College of Agriculture, Portugal, GPS coordinates 40.211439, −8.451251. After collection, its aerial parts were dried in a ventilated incubator at 35 °C for 3 days and kept in a dark room until use.

### 3.3. Extraction of Phenolic Compounds

The extraction of phenolic compounds was performed according to the method described by Ferreira et al. [38] with adaptations. Briefly, 0.5 mm mesh powder of the aerial parts (flowers, leaves and stems) of *S. africana, S. officinalis ‘Icterina’* and *S. mexicana* (5 g) was extracted for 15 min using a decoction 1:20 (5 g in 100 mL of water), filtrated and concentrated using a rotary evaporator (BUCHI Labortechnik AG, Flawil, Switzerland). The resulting filtrated solution was concentrated in a rotary evaporator at 37 °C, followed by defatting with *n*-hexane (1:1 *v*/*v*). The resulted fraction was frozen, freeze-dried and kept under vacuum in a desiccator in the dark, for subsequent use. This procedure was performed as three independent assays.

### 3.4. Identification and Quantification of Phenolic Compounds

The total phenolic content of each *Salvia* extract was determined according to the adapted Folin–Ciocalteu colorimetric method and expressed as µg of gallic equivalents (GAE) per mg of extracts, as described by Pereira et al. [39]. The phenolic profile of the aqueous extracts was determined by liquid chromatography analysis using an Ultimate 3000 (Dionex Co., San Jose, CA, USA) apparatus equipped with an ultimate 3000 Diode Array Detector (Dionex Co., San Jose, CA, USA) and a Thermo LTQ XL mass spectrometer (Thermo Scientific, San Jose, CA, USA), following a method described by Afonso et al. [40]. Gradient elution was carried out with a mixture of 0.1% (*v*/*v*) of formic acid in water (solvent A) and acetonitrile (solvent B). The solvent gradient used consisted of a series of linear gradients starting from 5% of solvent B and increasing to 23% at 14.8 min, to 35% at 18 min, and to 100% at 21 min over three minutes, followed by a return to the initial conditions. Operations of the mass spectrometer were carried out using the conditions previously described [41].

Rosmarinic acid and other less expressive compounds (*trans*-5-*O*-caffeoylquinic acid, caffeic acid and salvianolic acid B) were identified using standard commercial compounds. In cases that standard commercial compound was not available (e.g., yunnaneic acid E), identification of the phenolic component was based on the interpretation of ultraviolet (UV) spectral and mass spectrometry (MS) data, plus comparison to literature. Quantification was performed by the external standard method using the calibration curves of structurally related standard compounds. Rosmarinic acid was used to quantify rosmarinic acid and caffeoylrosmarinic acid isomers; 5-*O*-caffeoylquinic acid was used to quantify caffeoylquinic acids while other caffeic acid derivatives were quantified as equivalents of caffeic acid; salvianolic acid B was used to quantify salvianolic acid B isomers, yunnaneic acid isomers and sagerinic acid isomers; 2,4-dimethyl benzoic acid was quantified with 4-*O*-hydroxybenzoic acid; ferulic acid derivatives and coumaric acid derivatives were quantified as equivalents of ferulic acid and 4-*O*-coumaric acid, respectively; luteolin glycosides, apigenin and its glycosides, and hesperidin, were quantified as equivalents of luteolin-7-*O*-glucoside, apigenin-7-*O*-glucoside and eriodictyol-7-*O*-glucoside, respectively; Depending on the UVmax of each compound, the quantification was performed at 280, 320, or 340 nm, also considering the limit of detection (LOD) and limit of quantification (LOQ). LOD and LOQ were determined from the parameters of the calibration curves, being defined as 3.3 and 10 times the value of the regression error divided by the slope, respectively [40].

### 3.5. Antibacterial Activity

The antibacterial potentials of the *S. africana, S. officinalis ‘Icterina’* and *S. mexicana* species were evaluated against five bacterial strains, including Gram-positive bacteria (*S. epidermidis* NCTC 11,047 and *S. aureus* NCTC 6571) and Gram-negative bacteria (*S. typhimurium* NCTC 12023, *E. coli* NCTC 9001, and *P. aeruginosa* NCTC 10662) from the National Collection of Type Cultures (NCTC), operated by Public Health England, Salisbury, United Kingdom. All strains were cultured in Mueller-Hinton agar and incubated at 37 °C for 24 h. The minimum inhibitory concentration (MIC) and minimum bactericidal concentration (MBC) of aqueous solutions of three *Salvia* species were determined by the broth microdilution method using an adapted method previous described by Afonso et al. [40]. MIC is defined as the lowest concentration at which visible growth is inhibit, while MBC is the lowest concentration of the tested substance which has a bactericidal effect. Briefly, bacterial suspensions were prepared by direct colony suspensions and a final inoculum of 1.5 × 10^5^ colony forming units (CFU)/mL was required for final suspensions that was diluted in a 1:100 ratio in Mueller-Hinton broth. Next, 100 µL of this medium was dispensed into wells of 96-well micro titer plates and *Salvia* aqueous extracts were added and serially diluted four times across the plate. Then, 100 µL of bacteria suspension was added to each well and the plates were incubated at 37 °C for 24 h. The assay for each pathogen was repeated three times. MBC values are determined by sub-culturing from each negative well onto Mueller–Hinton agar and confirmation the lowest concentration with no visible growth [42]. Nisin was used as the positive control.

### 3.6. Antioxidant Activity

#### 3.6.1. 2,2-Diphenyl-1-Picrylhydrazyl Radical (DPPH^•^) Scavenging Test

The extracts capacity (0.002–0.02 mg/mL) for scavenging DPPH^•^ was evaluated following the procedure previously described by Catarino et al. [43]. Ascorbic acid was used as positive control and the results were expressed as EC_50_ values (sample concentration providing 50% of antioxidant activity, i.e., concentration of the extract able to inhibit the 50% of the DPPH radical).

#### 3.6.2. Reducing Power Test

The ability of the three sage extracts to reduce iron(III) to iron(II) was carried out according to a procedure described before [43], in which the antioxidant compounds from the extracts (0.008–0.045 mg/mL) form a colored complex with potassium ferricyanide, trichloroacetic acid, and ferric chloride, measurable at 700 nm. BHA (butylated hydroxyanisole) was used as positive control and the results were expressed as EC_50_ values, corresponding to 0.5 of absorbance.

#### 3.6.3. Thiobarbituric Acid Reactive Substances (TBARS)

The TBARS assay was evaluated for the extracts (0.0195–0.625 mg/mL) by the inhibition of lipid peroxidation in porcine (*Sus scrofa*) brain homogenates, according to a procedure described by Martins et al. [44]. The color intensity of the malondialdehyde-thiobarbituric acid (MDA-TBA) was measured as its absorbance at 532 nm; the inhibition ratio (%) was calculated using the following equation: [(A − B)/A] × 100%, where A and B were the absorbance of the control and the sample solutions, respectively. Trolox was used as positive control and the results were expressed as EC_50_ values, i.e., concentration of the extract able to inhibit peroxidation by 50%.

#### 3.6.4. β-Carotene Bleaching Inhibition Assay

β-carotene linoleate general assay was performed as described by Barros et al. [45], evaluating the β-carotene bleaching inhibition capacity of samples (0.039–0.312 mg/mL). Trolox was used as positive control and the results were expressed as EC_50_ values i.e., concentration of the extract able to inhibit peroxidation by 50%.

### 3.7. Anti-Inflammatory Activity

The extracts ability in scavenging the NO was evaluated in the mouse macrophage-like cell line RAW 264.7 following the general procedure previously described [46]. Cells were treated under 5% CO_2_ in humidified air, using DMEM culture medium enriched with 10% heat inactivated fetal bovine serum, glutamine, and antibiotics at 37 °C. For the tests, cells were seeded in 96-well plates (150,000 cells/well) and allowed do attach to the plate overnight. Then, these were treated with extract solutions (concentration between 25 and 100 µg/mL, for each extract) for 1 h, followed by the stimulation with LPS (1 µg/mL) for 18 h. The effect of all the tested samples in the absence of LPS was also evaluated, in order to observe if they induced changes in NO basal levels. Dexamethasone (50 µM) was used as a positive control while negative controls had no added LPS. The NO levels produced were determined by the Griess reaction, used for measuring the nitrite accumulation in the culture supernatant on macrophage cell line RAW 264.7 [46]. The anti-inflammatory activity of each extract was determined by calculating EC_50_ values (μg/mL), which correspond to the sample concentration that provides 50% inhibition of NO production.

### 3.8. Cytotoxicity in Tumor Cell Lines and Primary Porcine Liver Cells

The cytotoxic effect of the three *Salvia* species towards four human tumor cell lines were carried out by the sulforhodamine B (SRB) assay, using the conditions established by Souza et al. [46]. The tumor cell lines MCF-7 (breast adenocarcinoma), NCI-H460 (non-small cell lung cancer), HeLa (cervical carcinoma), and HepG2 (hepatocellular carcinoma), were maintained in enriched medium, at 37 °C, in a humidified air incubator containing 5% CO_2_. Each cell line was plated at an appropriate density (7.5 × 10^3^ cells/well for MCF-7 and NCI-H460 or 1.0 × 10^4^ cells/well for HeLa and HepG2) in 96-well plates. The cytotoxicity of the sage extracts were also tested by hepatotoxicity assay, in a primary non-tumor cell culture obtained from porcine liver (PLP2) following the procedures described [46]. For the tests, cells were seeded (at 1.0 × 10^4^ cells/well) in enriched medium. The cytotoxicity results were expressed as GI_50_ values (µg/mL), corresponding to sample concentration that inhibited 50% of the cell growth. Ellipticine was used as positive control.

### 3.9. Statistical Analysis

The results were analyzed using GraphPad Prism 6 (GraphPad Software, San Diego, CA, USA). Data were expressed as mean ± S.D. of 3–4 independent experiments performed at least in triplicate. One-way analysis of variance (ANOVA) followed by Tukey’s test was used to detect any significant differences among different means. The *p*-value less than 0.05 were assumed as significant difference.

## 4. Conclusions

The phenolic composition of *S. africana*, *S. officinalis ‘Icterina’* and *S. mexicana* aqueous extracts were assessed and their biological benefits evaluated. The three aqueous extracts were mainly characterized by distinct amounts of phenolic acids and flavonoids, which can be hypothesized to be partially related to their biological activities. In particular, one can emphasize the antioxidant, anti-inflammatory and cytotoxic potentials of *S. africana*. The aqueous extract of this species exhibited similar potency to that of positive controls in DPPH^•^, ferric reducing power and TBARS methods, and was also promising regarding the ability to inhibit the production of NO by LPS-stimulated RAW 264.7 macrophages cells (EC_50_ = 47.8 ± 2.1 μg/mL). Moreover, it showed a tendency to high cytotoxicity towards tumoral cells (hepatocellular HepG2, cervical HeLa and breast MCF-7 carcinoma cell lines) when compared to normal cells, underlining its probable selectivity. In turn, consistent with the low phenolic content in *S. mexicana* aqueous extract, this was the least promising regarding most activities, a fact that could reinforce the involvement of phenolic compounds in such effects. *S. officinalis ‘Icterina’* aqueous extract presented high content of glyosidic flavones and exhibited antibacterial potential with inhibitory and lethal effect against Gram-positive (MIC and MBC of 0.94–3.75 mg/mL) and Gram-negative bacteria (MIC and MBC in the range 3.75–7.5 mg/mL). Overall, this study suggests health-promoting effects from *Salvia* aqueous extracts, particularly for *S. africana*, albeit further investigation is needed using cellular/*in vivo* models, in order to prove this hypothesis and to elucidate their targets of inhibition on events of oxidation, inflammatory and apoptotic or necrotic death.

## Figures and Tables

**Table 1:** Identification and quantification of phenolic compounds from *S. africana, S. officinalis ‘Icterina’* and *S. mexicana* aqueous extracts.

RT	UV_max_	[M − H]^−^	MS ^2^ Main Fragments	Probable Compound	S. afr *	S. ict *	S. mex *
1.5	275	149	103, 87, 131, 59	2,4-DimethylBA	3.4 ± 0.2	4.7 ± 0.1	5.1 ± 0.1
1.7	205	191	111, 173	Quinic Ac	D	0.40 ± 0.05	D
3.6	280	197	179, 73, 153	Danshensu	6.5 ± 0.3	D	3.5 ± 0.1
4.2	278	315	153	Protoc Ac Hex	-	-	D
5.0	290 sh, 324	353	191, 179, 135	*cis*-3-*O*-CQA	-	-	3.8 ± 0.0
5.3	290, 327	311	149, 179	Caftaric Ac	-	-	3.50 ± 0.04
8.3	313	295	163	*p*-Coum Ac Pent	-	-	0.03 ± 0.01
8.8	290 sh, 325	353	191	*trans*-5-*O*-CQA	-	-	1.70 ± 0.06
9.4	ND	325	163, 119	Caff Hex	-	-	0.3 ± 0.0
9.5	290 sh, 325	353	173, 179, 191	4-*O*-CQA	-	-	2.5 ± 0.3
9.7	290 sh, 323	179	135	Caff Ac	2.3 ± 0.1	2.50 ± 0.04	0.9 ± 0.1
11.7	271	1077	358, 179, 296, 494	Galotannin Der	D	-	-
12.1	271, 336	593	473, 503, 353	Api-6-*C*-Glc-7-*O*-Glc	-	3.5 ± 0.1	-
12.3	289, 329	295	207, 179, 133, 135	Caff Malic Ac	-	-	1.00 ± 0.03
12.6	286, 320	313	269, 179, 135	SA F	-	2.7 ± 0.2	D
13.1	291, 311	637	351, 285, 193	Ferulic Ac Der	-	0.6 ± 0.1	-
13.5	274	571	527, 483, 439, 373	YA E (isomer 1)	3.8 ± 0.3	-	4.4 ± 0.2
13.9	256, 267 sh, 345	447	327, 357	Lut-*C*-Hex	-	-	4.1 ± 0.2
13.9	281, 345	477	301, 373, 343, 397	Hydroxy-Lut-GlcA	D	2.90 ± 0.05	-
14.1	276	571	527, 439, 553, 483	YA E (isomer 2)	5.2 ± 0.7	-	-
14.3	276	597	579, 355, 312, 295, 197, 179	YA F	8.7 ± 0.9	-	-
14.7	274	571	527, 509, 553, 483, 285	YA E (isomer 3)	18.3 ± 0.9	-	2.80 ± 0.01
14.8	267, 345	621	351, 269	Api-diGlcA	-	D	-
15.1	276	555	313, 357	SA K	3.9 ± 0.3	-	-
15.4	274	571	527, 553, 509, 329	YA E (isomer 4)	D	-	-
15.6	235, 275, 320	539	297, 359, 495, 279	YA D (isomer 1)	D	-	-
15.9	280, 333	461	285	Scut-*O*-GlcA	-	9.7 ± 0.2	-
15.9	277	539	341, 253, 315, 359	YA D (isomer 2)	2.4 ± 0.2	-	3.5 ± 0.1
16.1	255, 266 sh, 345	461	285	Lut-7-*O*-GlcA	18.7 ± 1.2	18.2 ± 0.4	-
16.5	268	571	527, 409	YA E (isomer 5)	30.8 ± 1.7	-	-
17.2	278	717	519, 475, 537, 339	SA B (isomer 1)	-	-	1.50 ± 0.02
17.3	279	571	527, 553, 329	YA E (isomer 6)	15.4 ± 0.84	-	-
17.6	283	719	359, 539, 521, 341	Sag Ac (isomer 1)	6.0 ± 0.3	9.1 ± 0.4	1.20 ± 0.01
18.1	269, 329	431	269	Api Hex	-	1.70 ± 0.03	-
18.3	238, 341	607	299, 284	Chrys Rut	-	-	D
18.4	267, 337	445	269, 175	Api-*O*-GlcA	4.6 ± 0.2	32.8 ± 0.5	-
18.6	270	717	555, 519, 475, 357	SA B (isomer 2)	3.2 ± 0.2	3.3 ± 0.2	-
18.7	284, 330 sh	609	301	Hesperidin	-	-	0.50 ± 0.03
19.0	290 sh, 328	359	161, 179, 197, 223	RA	77.0 ± 3.6	52.7 ± 0.5	29.4 ± 0.6
19.2	282 sh, 327	537	493, 295	Caff RA (isomer 1)	3.0 ± 0.2	-	-
19.5	285 sh, 305	537	493, 295	Caff RA (isomer 2)	14.2 ± 1.5	-	1.0 ± 0.1
19.8	278	719	521, 341, 359	Sag Ac (isomer 2)	-	-	2.2 ± 0.1
21.4	285sh, 330	537	456, 493, 375, 359	Caff RA (isomer 3)	2.5 ± 1.0	-	-
				Sum	231.6 ± 7.5	144.1 ± 2.7	72.8 ± 0.7
			Total Phenolic Content ^1^		350.6 ± 14.9	229.0 ± 44.0	158.9 ± 38.0

Mean values ± S.D.; * Values are expressed as μg/mg extract; ^1^ Determined by Folin–Ciocalteu assay and expressed as µg of gallic equivalents per mg of extract; D—Detected; Ac—Acid; Api—Apigenin; BA—Benzoic acid; Caff Ac—Caffeic acid; Caff—Caffeoyl; CQA—Caffeoylquinic acid; Chrys—Chrysoeriol; Coum—Coumaroyl; D—Detected; Der—Derivative; Glc—Glucoside; GlcA—Glucuronide; Hex—Hexoside; Lut—Luteolin; ND—Not determined; Pent—Pentoside; Protoc—Protocatechuic; Rut—Rutinoside; RA—Rosmarinic acid; RT—Retention time; Sag—Sagerinic; SA—Salvianolic acid; *S. afr*—*S. africana*; *S. ict*—*S. officinalis ‘Icterina’*; *S. mex*—*S. mexicana*; Scut—Scutellarein; sh—shoulder; UV – Ultraviolet; YA—Yunnaneic acid.

**Table 2:** Antibacterial properties (MIC/MBC, mg/mL) of *S. africana*, *S. officinalis ‘Icterina’* and *S. mexicana* aqueous extracts against selected test bacteria.

Bacteria	*S. africana*	*S. officinalis ‘Icterina’*	*S. mexicana*	Nisin
*Staphylococcus aureus*	0.63/1.25	0.94/0.94	1.19/1.19	<0.63/<0.63
*Staphylococcus epidermidis*	1.25/1.25	3.75/3.75	4.75/9.50	<0.63/<0.63
*Salmonella typhimurium*	5.0/5.0	3.75/3.75	>9.50/>9.50	0.50/0.50
*Escherichia coli*	10.0/10.0	7.5/7.5	9.50/9.50	0.50/1.0
*Pseudomonas aeruginosa*	>10.0/>10.0	7.5/7.5	9.50/9.50	1.0/1.0

MIC- minimum inhibitory concentration; MBC- minimum bactericidal concentration.

**Table 3:** Antioxidant, anti-inflammatory and cytotoxic properties of *S. africana*, *S. officinalis ‘Icterina’* and *S. mexicana* aqueous extracts.

Assays	*S. africana*	*S. officinalis ‘Icterina’*	*S. mexicana*	Standard
**Antioxidant Activity** (EC_50_, μg/mL)				
DPPH^•^	6.6 ± 0.7 ^b^	10.4 ± 0.2 ^a^	10.0 ± 1.1 ^a^	6.68 ± 0.7 ^b^
Ferric reducing power	21.2 ± 2.7 ^b^	42.3 ± 3.1 ^a^	34.0 ± 6.5 ^a^	16.1 ± 2.0 ^b^
TBARS inhibition	21.0 ± 0.3 ^c^	23.0 ± 0.2 ^b^	26.2 ± 0.9 ^a^	23.0 ± 1.0 ^b^
β-Carotene bleaching inhibition	128.6 ± 6.3 ^c^	146.6 ± 7.0 ^b^	164.6 ± 7.7 ^a^	41.7 ± 0.3 ^d^
**Anti-Inflammatory Activity** (EC_50_, μg/mL)				
NO production inhibition	47.8 ± 2.1 ^b^	60.3 ± 1.5 ^a^	66.3 ± 5.4 ^a^	16.0 ± 1.0 ^c^
**Cytotoxic activity** (GI_50_, μg/mL)				
HepG2 (hepatocellular carcinoma)	42.5 ± 4.2 ^a^	48.9 ± 4.4 ^a^	52.4 ± 4.9 ^a^	1.0 ± 0.2 ^b^
HeLa (cervical carcinoma)	58.8 ± 4.5 ^b^	89.2 ± 7.2 ^a^	61.0 ± 5.6 ^b^	2.0 ± 0.1 ^c^
MCF-7 (breast carcinoma)	61.3 ± 9.8 ^a^	71.0 ± 3.4 ^a^	66.2 ± 4.6 ^a^	1.0 ± 0.04 ^b^
NCI-H460 (non-small cell lung cancer)	286.6 ± 17.6 ^a^	273.3 ± 14.3 ^a^	257.6 ± 21.4 ^a^	1.0 ± 0.1 ^b^
PLP2 (non-tumour cells)	336.4 ± 10.8 ^a^	304.9 ± 11.1 ^a^	296.8 ± 7.3 ^a^	3.0 ± 1.0 ^b^

DPPH^•^—2,2-diphenyl-1-picrylhydrazyl radical; NO—nitric oxide; TBARS—thiobarbituric acid reactive substances. Ascorbic acid and butylated hydroxyanisole (BHA) were used as reference compounds in DPPH and ferric reducing power assays, respectively; Trolox was used as reference compound in the both TBARS and β-carotene inhibition methods; Dexamethasone and ellipticine were used as reference compounds in anti-inflammatory and cytotoxicity activities, respectively. Means followed by the same letters (a, b, c, d) in rows do not differ by Tukey’s test (*p* > 0.05).
